# Supplementary material for: SyNDI: synchronous network data integration framework
Source: BMC Bioinformatics. 2018 Nov 6;19:403. doi: 10.1186/s12859-018-2426-5 (PMC6219086; doi:10.1186/s12859-018-2426-5)
Supplement: Supplementary file 5 — Files of the example presented in Additional file 4. This additional file is a zip package that contains all files used in Additional file 4. Detailed descriptions of each of these files are presented in a README file included in this zip package. (GZ 30411 kb) [file 12859_2018_2426_MOESM5_ESM.gz › SyNDITools/Meme2Fimo/meme_4.9.0_4.tar.gz!/meme_4.9.0/doc/purge.html]

Purge Manual


# Purge Manual

Purge removes redundant sequences from a FASTA file. This is
recommended in order to prevent highly similar sequences distorting the
search for motifs. Purge was written by Andy Neuwald and is described in
more detail in Neuwald *et al.*, "Gibbs motif sampling: detection
of bacterial outer membrane protein repeats", Protein Science, **4**:1618--1632,
1995.

Purge works with either DNA or protein sequences and creates an
output file such that no two sequences have a (gapless) local alignment
score greater than a threshold specified by the user. The alignment
score is based on the BLOSUM62 matrix for proteins, and on a +5/-1
scoring scheme for DNA. Purge can also be used to mask tandem repeats.
It uses the XNU program for this purpose.

## Using Purge

- For typical-length protein sequences, a reasonable purge
  threshold is around 150. To purge a file of sequences named
  "examples/lipocalin.s", do the following command. The purged sequences
  will be put in file "examples/lipocalin.s.b150". You should experiment
  with different purge thresholds depending on your particular needs.
  Depending on the application values between 60 and 250 may be
  appropriate.

  ```
    purge examples/lipocalin.s 150
  ```
- For DNA sequences of about 100 bp, a reasonable purge
  threshold is about 175. As the sequences become longer, higher
  thresholds will be required to avoid removing too many sequences. To
  purge a file of sequences named "examples/crp0.s", do the following
  command. The purged sequences will be put in file
  "examples/crp0.s.b175".

  ```
    purge examples/crp0.s 175
  ```

## Purge Usage Message

- If you type "purge" at the command line, you will receive the
  following usage message.

```
usage: purge file score 
  options:
     [-n]    - sequences are DNA (default: protein)
     [-b]    - use blast heuristic method (default for protein)
     [-e]    - use an exhaustive method (default for DNA)
     [-q]    - keep first sequence in the set
     [-x]    - use xnu to mask protein tandem repeats

  Purge creates an output file from the input file such that
  no two sequences have local alignment score greater than .
  The output file is named ..
  Substitution matrices: BLOSUM62 (protein), +5/-1 (DNA).
```
